# SENSE OF COHERENCE AND REHABILITATION OUTCOMES: A 3-YEAR LONGITUDINAL STUDY

**DOI:** 10.2340/jrm.v58.46229

**Published:** 2026-09-29

**Authors:** Anne Mette BERGET, Øystein HETLEVIK, Merethe HUSTOFT, Jörg ASSMUS, Vegard Pihl MOEN

**Affiliations:** 1 Centre of Habilitation and Rehabilitation in Western Norway, Haukeland University Hospital, Bergen; 2 Department of Global Public Health and Primary Care, University of Bergen, Bergen; 3 Department of Health and Functioning, Western Norway University of Applied Sciences, Bergen; 4 Centre for Clinical Research, Haukeland University Hospital, Bergen, Norway

**Keywords:** rehabilitation, sense of coherence, coping resources, health, functioning, patient-reported outcomes

## Abstract

**Objectives:**

To examine Sense of Coherence over a 3-year period and investigate whether baseline levels and changes in Sense of Coherence are associated with rehabilitation outcomes.

**Design:**

Longitudinal cohort study.

**Participants:**

Patients accepted for specialized rehabilitation (*n* = 601).

**Methods:**

Sense of Coherence and rehabilitation outcomes, defined by health and functioning were assessed before rehabilitation and after 3 years after using the 13-item Sense of Coherence questionnaire, the EuroQol visual analogue scale, and the Medical Outcomes Study Short Form 36. Sense of Coherence was analysed descriptively, and associations with rehabilitation outcomes were examined using analysis of covariance, adjusted for age, sex, level of education, referral diagnosis, and multimorbidity.

**Results:**

Participants reported lower Sense of Coherence, health, and functioning compared with reference populations. Mean change in Sense of Coherence over 3 years was small at the group level, at 0.7 (SD 9.7) points. Substantial variation was observed in both baseline Sense of Coherence and individual change over time. Both baseline Sense of Coherence and change in Sense of Coherence were independently associated with rehabilitation outcomes, with stronger associations observed for overall health and mental functioning than for physical functioning.

**Conclusion:**

These findings suggest that interventions aimed at strengthening Sense of Coherence may improve rehabilitation outcomes across patient groups.

## LAY ABSTRACT

This study explored how patients’ Sense of Coherence (a coping resource), develops over 3 years following specialized rehabilitation. We also examined whether changes in Sense of Coherence were linked to rehabilitation outcomes, defined by health and daily functioning. A total of 601 patients completed questionnaires before specialized rehabilitation and again 3 years later. Participants reported weaker Sense of Coherence and poorer health and daily functioning than people in the general population. Changes in Sense of Coherence over time were small overall, but individual changes varied widely. Both a stronger Sense of Coherence at the start of rehabilitation and improvements over time were linked to better rehabilitation outcomes, particularly for overall health and mental functioning. These findings suggest that rehabilitation programmes may benefit from helping patients strengthen their Sense of Coherence, which could support better recovery and well-being across different patient groups.

Empowering individuals to take control of their emotions and actions related to their health issue, and equipping them with effective coping strategies to manage stress, are significant aspects of rehabilitation (1). Within the context of rehabilitation as a healthcare service, an individual’s life orientation may undergo significant changes influenced by interventions, individual coping strategies, and evolving perceptions of one’s health and functioning, which are the main outcomes of rehabilitation (2).

The psychosocial aspects of health are well integrated in rehabilitation, recognizing the interplay between an individual’s resources, and their capacity to improve mental and physical functioning (3). One psychological construct that has gained attention in recent decades is the Sense of Coherence (SOC), which refers to a person’s view of life and their ability to respond to stressful situations (4). SOC encompasses an individual’s belief in their comprehension, sense-making of life events, and challenges, as well as their ability and motivation to effectively manage and cope with them. Consequently, a stronger SOC may be a significant factor in achieving better health outcomes along a rehabilitation trajectory.

Synthesized research and recent studies show that SOC might be changeable (5–7), challenging the theory proposed by the founder of SOC, Aaron Antonovsky. He posited that SOC is relatively stable after the age of 30 (4). However, it is found that interventions may strengthen SOC among individuals with various diagnoses (8–11).

It is hypothesized that individuals with a well-developed SOC (strong SOC) display a more pronounced inclination to identify and resolve challenges than those with a less developed SOC (weak SOC) (12). In a prospective cohort study the greatest improvement in SOC (46–58%) was observed amongst participants with a strong SOC at baseline (13). As SOC may directly impact both health and functioning (14), individuals with a stronger SOC may be better able to engage in rehabilitation, utilize coping resources, and adapt to health-related challenges. Consequently, a strong baseline SOC may contribute to better health and functioning over time. However, the relationship may also operate in the opposite direction, as improvements in health and functioning achieved during rehabilitation may strengthen an individual’s sense of manageability, meaningfulness, and control. Thus, the relationship between SOC and rehabilitation outcomes may be dynamic and potentially bidirectional. Examining both baseline SOC and changes in SOC may therefore provide a more comprehensive understanding of long-term rehabilitation outcomes than considering baseline SOC alone.

Studies investigating changes in SOC over time commonly have a follow-up period of up to 1 year. Few studies have examined changes in SOC over longer periods. The study by Gunnarsson and Bjørklund had a 3-year follow-up; however, it included a relatively small sample size (*n* = 31) (15).

It is challenging to determine the duration of rehabilitation effects, as it varies across numerous outcome measures. While studies indicate that behavioural interventions may have effects lasting up to 48 months (16), less is known about whether changes in SOC remain stable or whether individuals change their initial SOC level during the rehabilitation trajectory (17).

Furthermore, while existing literature recognizes the significance of SOC across various health-related areas and its association with certain outcomes, there remains a significant gap in understanding the association between SOC, changes in SOC, and rehabilitation outcomes. In our previous research, we have found that baseline SOC can predict change in functioning after rehabilitation (18). While baseline SOC provides essential initial insight, examining changes in SOC during the rehabilitation trajectory is likely to yield a more comprehensive and accurate prediction of rehabilitation outcomes at 3-year follow-up. Such an approach not only underscores the ongoing development of personal resources throughout rehabilitation but also aligns with the dynamic, transformative goals in rehabilitation.

Thus, the aim of this study is to investigate SOC in a rehabilitation population. First, we examine SOC at baseline, before rehabilitation, and changes in SOC over a 3-year trajectory. Second, we investigate whether baseline SOC and changes in SOC are independently associated with rehabilitation outcomes defined by health and functioning at 3-year follow-up.

## METHODS

This study is a part of the Rehabilitation Cohort Western study (REKOVE), a longitudinal study in Western Norway. REKOVE invited all patients in the Western Norway Health Region who were accepted for inpatient or outpatient rehabilitation at a specialized healthcare rehabilitation centre between January 2015 and June 2015. The study collected both patient-reported data (2015, 2016, and 2018) and register data (2010–2020). The study and study participants are described thoroughly in previous papers (18–20), including flowcharts of participants.

Participants who responded to the 13-item Sense of Coherence (SOC-13) questionnaire at baseline and at the 3-year follow-up were included in this study.

### Variables

*Predictors:* SOC was assessed at baseline (before rehabilitation) and again 3 years later using the 13-item SOC-13. Participants rated items on a 7-point Likert scale ranging from “never” (1) to “very often” (7). A total score between 13 and 91 was calculated, with higher scores indicating a better SOC. The instrument has shown sound psychometric properties (21, 22).

The minimal clinically important change (MCID) for SOC has not been established. To aid interpretation of changes in SOC, we applied the half-standard deviation criterion proposed by Norman et al. as a distribution-based approach (23).

*Outcomes:* Rehabilitation outcomes were measured with general self-reported health and physical and mental functioning.

The EuroQoL EQ-5D-5L visual analogue scale (EQ-VAS) was used to measure general self-reported health. EQ-VAS provides a subjective assessment of health status. The participants assess their general health on a scale from 0 (worst) to 100 (best). The EQ-5D-5L has been validated across various populations and settings (24-27). Moreover, the EQ-5D-5L is to be implemented in specialized rehabilitation in Norway.

Physical and mental functioning were measured using the Medical Outcomes Study Short Form (SF-36), version 1. In SF-36, 8 functional domains are measured and summarized into 2 components: (*i*) the physical component summary (PCS) measuring general health, bodily pain, physical functioning, and role physical, and (*ii*) the mental component summary (MCS) measuring vitality, social functioning, mental health, and role emotional (28, 29). According to the SF-36 scoring manual, both PCS and MCS scores were calculated on a scale from 0 to 100, with a higher score indicating better functioning (28). To obtain PCS and MCS scores, participants had to respond to at least 50% of the items (28). The SF-36 has been found to be a valid and reliable instrument (30).

*Covariates:* The referral diagnoses, based on the International Classification of Diseases, Tenth Revision (ICD-10), were used to categorize patients into 5 diagnostic groups: musculoskeletal, neoplasms, cardiovascular, neurology, and other.

Data on multimorbidity were obtained from the Norwegian Control and Payment of Health Reimbursements Database (KUHR). All general practitioners (GPs) in primary health care in Norway have municipal operating agreements and receive part of their payment through fee‑for‑service, submitting claims for each patient contact. These claims are stored in KUHR. As part of each claim, GPs assign a diagnosis using the International Classification of Primary Care, version 2 (ICPC‑2). A selection of diagnoses based on the ICPC‑2 multimorbidity index was used to categorize multimorbidity as “0”, “1–2”, or “3 or more” chronic conditions registered during a 3‑year period prior to specialized rehabilitation (31).

Age was calculated using the baseline year, sex was categorized as male and female, and the highest level of education completed was categorized as primary school (at most 10 years), upper secondary school (11–13 years), or university (≥14 years) and marital status was categorized as unmarried, married, widow(er), and divorced.

### Statistics

Descriptive statistics using number, percentage, mean, and standard deviation are used to describe the study cohort and SOC (Fig. 1 and Tables I, II).

**Fig. 1:**
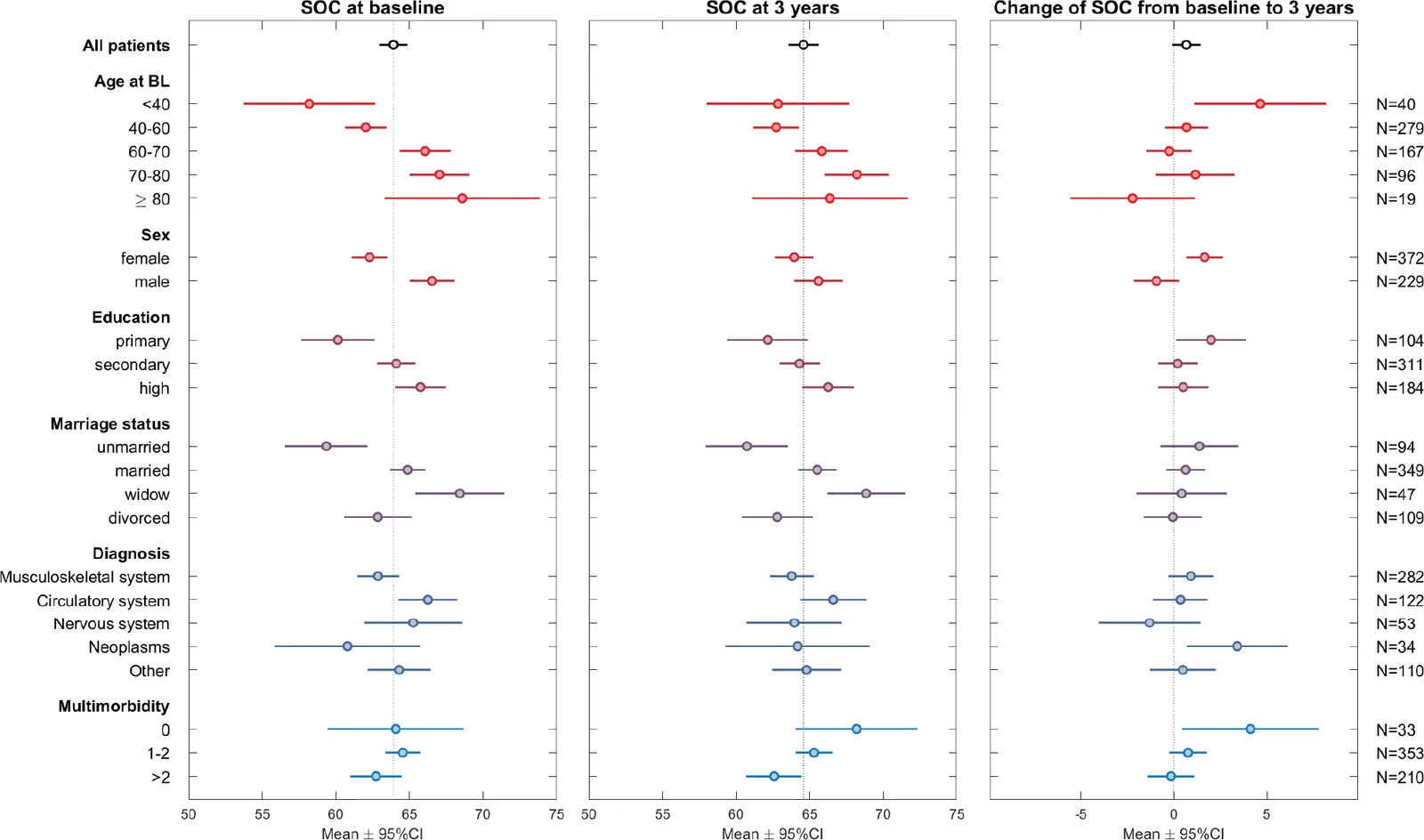
Sense of Coherence (SOC) at baseline, 3 years, and change scores for the total study cohort and by age groups, sex, level of education, marital status, referral diagnoses, and multimorbidity. 95% CI = 95 % confidence interval.

**Table I:** Characteristics of the study population (*n* = 601)

Characteristic	*n*	*n* = 601
Age at baseline (years)	601	59 (50, 68)
Sex	601	
Female		372 (62%)
Male		229 (38%)
Level of education		
Primary school		104 (17%)
Upper secondary school		311 (52%)
University		184 (31%)
Marital status	599	
Unmarried		94 (16%)
Married		349 (58%)
Widow		47 (7.8%)
Divorced		109 (18%)
Referral diagnoses		
Musculoskeletal		282 (47%)
Circulatory		122 (20%)
Neurology		53 (8.8%)
Neoplasms		34 (5.7%)
Other		110 (18%)
Multimorbidity	596	
0		33 (5.5%)
1–2		353 (59%)
>2		210 (35%)
SOC-13 baseline	601	65 (56, 73)
EQ-VAS baseline	593	50 (40, 65)
SF36-MCS baseline	558	46 (36, 54)
SF36-PCS baseline	558	32 (26, 39)

Median (Q1, Q3); *n* (%).

**Table II:** Sense of Coherence (SOC-13), Physical (PCS) and Mental (MCS) functioning (SF-36) and self-reported health (EQ-VAS) as reported by the participants (*n* = 601) before rehabilitation (baseline) and 3 years later

	SOC-13			EQ-VAS			SF-36					
							PCS			MCS		
	*n*	Mean (SD)	Min–max	*n*	mean (SD)	Min–max	*n*	Mean (SD)	Min–max	*n*	Mean (SD)	Min–max
Total study cohort												
Baseline	601	63.9 (12.1)	24–91	575	46.1 (18.1)	0–100	537	31.9 (9.1)	8.3–61.2	537	42.8 (12.2)	8.9–70.8
3-year follow-up	601	64.6 (12.7)	24–91	382	54.2 (20.1)	5–98	359	35.1 (10.6)	12.2–60.9	359	45.6 (11.6)	11.4–71.9

SOC-13: Sense of Coherence questionnaire (13-91, 91 best); SF-36: Medical Outcome Study Short Form; PCS: Physical Component Summary (scale 0–100, 100 best); MCS: Mental Component Summary (0–100, 100 best); EQ-VAS: EuroQol visual analogue scale (0–100, 100 best); SD: standard deviation.

The prediction model for SOC at baseline was done in 3 steps. In the first step we estimated the univariable model for each predictor variable (SOC baseline and change in SOC); in the second step we estimated the full model containing all predictor variables. In the final model we included all predictor variables that had a *p*-value less than 0.1 in at least 1 of the previous steps (Table SI).

Analysis of covariance (ANCOVA) was used to examine associations between SOC and patient-reported outcomes (EQ-VAS, PCS, MCS) at 3-year follow-up. ANCOVA was chosen because it allows comparison of follow-up outcomes while adjusting for baseline levels of the same measure. This approach reduces bias from regression to the mean and generally provides more precise estimates than simple change-score models, which can be affected by measurement error and baseline variability (32). With 2 time points, ANCOVA is a statistically powerful and efficient approach and was therefore considered the most appropriate of the available longitudinal methods.

ANCOVA models were built for each outcome (EQ-VAS, MCS, PCS); the corresponding baseline score was included as a covariate. SOC was represented by 2 variables: the baseline SOC score and the change in SOC over the 3-year period. This modelling strategy allowed estimation of the independent contributions of baseline SOC and SOC change to each outcome, while holding baseline outcome levels constant.

Three unadjusted ANCOVA models were specified for each outcome: Model 1 included baseline SOC, Model 2 included SOC change, and Model 3 included both baseline SOC and SOC change, with the baseline outcome score included in all models. The final adjusted models additionally controlled for age, sex, marital status, level of education, and referral diagnosis to account for potential confounding. Robust standard errors were applied to account for potential heteroscedasticity. Results are presented as regression coefficients (*b*), 95% confidence intervals (CI), and *p*-values (Table III).

**Table III:** Associations between Sense of Coherence (SOC) and patient reported outcomes (EQ-VAS, SF-36) 3 years following specialized rehabilitation

EQ-VAS (*n* = 585)												
Variable	Model 1. SOC baseline			Model 2. SOC change			Model 3. SOC change + SOC baseline			Final model *		

	Beta	95% CI	*p*-value	Beta	95% CI	*p*-value	Beta	95% CI	*p*-value	Beta	95% CI	*p*-value
Baseline Score (EQ-VAS)	0.55	0.47, 0.63	< 0.001	0.63	0.56, 0.71	< 0.001	0.54	0.46, 0.62	< 0.001	0.55	0.47, 0.63	< 0.001
SOC-13 baseline score	0.24	0.11, 0.37	< 0.001				0.41	0.28, 0.54	< 0.001	0.42	0.29, 0.55	< 0.001
SOC-13 change				0.47	0.32, 0.62	< 0.001	0.62	0.47, 0.78	< 0.001	0.59	0.44, 0.75	< 0.001

SF-36, Physical Component Summary (PCS, *n* = 529)												
Variable	Model 1. SOC baseline			Model 2. SOC change			Model 3. SOC change + SOC baseline			Final model *		

	Beta	95% CI	*p*-value	Beta	95% CI	*p*-value	Beta	95% CI	*p*-value	Beta	95% CI	*p*-value

Baseline Score (PCS)	0.71	0.63, 0.78	< 0.001	0.73	0.65, 0.80	< 0.001	0.70	0.63, 0.78	< 0.001	0.65	0.57, 0.73	< 0.001
SOC-13 baseline score	0.14	0.08, 0.20	< 0.001				0.18	0.11, 0.24	< 0.001	0.18	0.12, 0.25	< 0.001
SOC-13 change				0.06	-0.02, 0.14	0.113	0.14	0.06, 0.23	0.001	0.13	0.05, 0.22	0.002

SF-36, Mental Component Summary (MCS, *n* = 529)												
Variable	Model 1. SOC baseline			Model 2. SOC change			Model 3. SOC change + SOC baseline			Final model *		

	Beta	95% CI	*p*-value	Beta	95% CI	*p*-value	Beta	95% CI	*p*-value	Beta	95% CI	*p*-value

Baseline score (MCS)	0.43	0.34, 0.53	< 0.001	0.59	0.53, 0.65	< 0.001	0.37	0.29, 0.45	< 0.001	0.37	0.29, 0.45	< 0.001
SOC-13 baseline score	0.19	0.10, 0.28	< 0.001				0.38	0.29, 0.46	< 0.001	0.37	0.28, 0.46	< 0.001
SOC-13 change				0.41	0.33, 0.49	< 0.001	0.55	0.47, 0.65	< 0.001	0.55	0.47, 0.64	< 0.001

* Adjusted for age, sex, marital status, level of education, and diagnoses.

EQ-VAS: EuroQol visual analogue scale (0–100, 100 best); SF-36: Medical Outcome Study Short Form; PCS: Physical Component Summary (scale 0–100, 100 best); MCS: Mental Component Summary (0–100, 100 best); SOC-13: Sense of Coherence questionnaire (13–91, 91 best); 95% CI: 95 % confidence interval.

The data were analysed using Stata/SE 17 for windows (StataCorp LLC, College Station, TX, USA) and R 4.5.2 (R Foundation for Statistical Computing, Vienna, Austria).

## RESULTS

### Study population, SOC at baseline, and change in SOC

The study population included 601 patients referred to specialized rehabilitation The mean age was 59 years (range 50–68), 62% were women, 31% had university education, and 47% were referred to specialized rehabilitation due to musculoskeletal diagnoses (see Table I).

The mean (SD) scores of SOC and outcome measures (EQ-VAS, SF-36) at baseline and 3 years following rehabilitation are presented in Table II.

The mean change in SOC at the group level was 0.7 points (SD 9.7) and did not reach statistical significance (T = 1.69, df = 600, *p* = 0.09). However, as shown in Fig. 1, there was large variation in SOC at baseline and change in SOC within the cohort. Based on the half-standard deviation criterion proposed by Norman et al. (23), 32% had an improvement of /≥4.85 points, 43% remained stable, and 25% declined.

### Associations between SOC and rehabilitation outcomes

As indicated in Table III, baseline SOC and change in SOC were independently associated with rehabilitation outcomes in the final ANCOVA models. For EQ‑VAS and MCS, both baseline SOC and SOC change were consistently associated with higher outcome scores. For PCS, baseline SOC was consistently associated with PCS scores 3 years following rehabilitation, while SOC change was only significant when modelled together with baseline SOC.

## DISCUSSION

The aim of the study was to examine SOC in a 3-year rehabilitation trajectory, and to investigate its association with rehabilitation outcomes as defined by change in health and functioning. Our findings show that SOC changes were marginal at group level in a 3-year trajectory, but individual variation was substantial. We found that both baseline SOC and SOC change were independently associated with rehabilitation outcomes.

### Sense of Coherence at baseline and change over 3 years

The mean SOC at baseline among our study population is lower compared with European samples clustering between 65 and 71 points (33, 34). At group level in our study, the change in SOC over 3 years was small (+0.7 points). Similarly, minor overall change has been reported among patients with musculoskeletal pain in primary care over a 1-year follow-up period (35). Additionally, prior research examining various latent classes of SOC trajectories over a 5-year period found that most participants demonstrate an increasing trend in SOC development (13).

Even though there were large variations within our cohort, neither the referral diagnoses nor multimorbidity appeared to influence change in SOC in our study sample. This could indicate that, in a rehabilitation context, individual resources or contextual factors play a more prominent role in SOC trajectories than the medical diagnoses themselves.

### Association with rehabilitation outcomes

In our study, the associations between SOC and rehabilitation outcomes differed by outcome domain. For EQ‑VAS and MCS, change in SOC showed larger coefficients than baseline SOC, indicating a stronger association of within‑person changes in SOC with EQ‑VAS and MCS outcomes 3 years following rehabilitation. This pattern differed from PCS, for which baseline SOC showed a more dominant association. Although change in SOC was associated with PCS at 3 years when baseline SOC was simultaneously accounted for, the magnitude of the associations between SOC and PCS was smaller.

This finding is consistent with salutogenic theory, which posits that SOC primarily influences how individuals perceive, interpret, find meaning in, and manage life stressors (4); thereby it may exert a stronger effect on subjective and psychological dimensions of health than on physical functioning. Improvements in SOC may translate more directly into perceived health and mental functioning. However, the independent association between SOC change and PCS in the fully adjusted models suggests that greater improvements in SOC were associated with better physical functioning, albeit to a lesser extent.

Importantly, the associations between SOC change and rehabilitation outcomes were consistent across the range of initial SOC levels. This finding suggests that improvements in SOC are associated with better outcomes among individuals with both low and high baseline SOC, especially in terms of perceived health and mental functioning, whereas effects on physical functioning were smaller.

To contextualize the magnitude of the observed associations between change in SOC and rehabilitation outcomes we used the half-standard deviation of Norman et al. (23). In the present sample, half a standard deviation of SOC change corresponded to approximately 5 points. An improvement of this magnitude in SOC was associated with an estimated increase of approximately 3 points in MCS, which meets the commonly accepted MCID for the SF‑36 (36). For EQ‑VAS, a corresponding improvement of approximately 3 points represents a modest proportion of the reported MCID range of 7–11 points, suggesting a smaller but potentially meaningful contribution (37). The estimated improvement in PCS was small (approximately 0.6 points) and remained below the established MCID threshold for SF‑36.

Given the substantial variability in SOC change observed in this cohort, the precise clinical implications should be interpreted with caution. Nonetheless, the findings consistently indicate that patients who achieve greater improvements in SOC tend to report better outcomes in health and functioning following rehabilitation. Thus, the results suggest that SOC may represent a potentially relevant factor in rehabilitation trajectories. This interpretation is consistent with previous research demonstrating that individuals with stronger SOC report better health and demonstrate greater capacity to cope with their health condition (21). However, causal effects cannot be established from our study. The relationship between SOC and rehabilitation outcomes may be complex and potentially bidirectional.

### Strengths and limitations

Our study adopts a health service perspective focusing on current rehabilitation practices. The study population was recruited from specialized rehabilitation institutions operating under contracts with the regional health authority. Although these institutions are guided by a common regulatory framework, the Norwegian definition of rehabilitation, and principles of evidence-based practice, we did not have access to detailed information regarding the specific content, intensity, duration, or composition of rehabilitation interventions delivered to individual participants. Consequently, rehabilitation should be considered a “black box” in the present study. While the overall rehabilitation approach is likely to have been conceptually similar across institutions, variation in intervention components and treatment intensity may nevertheless have contributed to differences in both SOC trajectories and rehabilitation outcomes. This should be considered when interpreting the findings.

A strength of this study is the longitudinal design, embracing rehabilitation as a health service as it is. The 3-year follow-up provided invaluable insights into the dynamic nature of patient experiences and health outcomes, offering a more comprehensive understanding compared with short-term observations. However, extended durations introduce complexities, including changes in healthcare practices, patient lifestyles, and unforeseen events, which can confound results. For practical reasons, a data collection effort that encompasses all of these aspects would be too extensive. Only participants who completed both the baseline and 3-year follow-up questionnaires were included in the analyses. As data were not available for individuals who did not respond at follow-up, a comparison between responders and non-responders could not be performed. Consequently, the potential for attrition bias cannot be excluded. Participants who remained in the study may differ systematically from those lost to follow-up, which could influence the generalizability of the findings.

In this study, health was measured using the EQ-5D-5L instrument, due in part to results indicating a greater change when using the EQ-VAS compared with the WHODAS-36 (38). Additionally, Norwegian health authorities have mandated the inclusion of the EQ-5D-5L in a rehabilitation register within the specialized healthcare services. Our findings, derived from a heterogeneous group of rehabilitation patients and framed from a healthcare service perspective, have the potential to contribute valuable insights for the design of future research projects.

Strengths of this study include the use of robust statistical models (ANCOVA), adjustment for key covariates, and long‑term follow‑up. However, a methodological limitation is that baseline SOC and change in SOC are mathematically related, which may introduce some dependency between predictors and increase the risk of multicollinearity. However, sensitivity analyses produced similar results, suggesting that this did not materially affect our findings.

However, causality cannot be inferred, and changes in SOC may reflect broader psychosocial adaptation processes that co‑occur with improvements in health and functioning. Additional limitations include reliance on self‑reported measures and the absence of an established minimal important change for SOC, which complicates interpretation of clinical relevance. This represents a limitation of SOC as an outcome and explanatory variable, as neither a theoretical nor an empirical basis for an absolute meaningful change threshold currently exists. Future research should therefore focus on elucidating mechanisms underlying SOC change and evaluating its potential as a target for intervention.

### Conclusion

There is sound evidence that baseline SOC can predict rehabilitation outcomes (18, 39, 40). Results from this study further highlight the importance of SOC as both a baseline resource and a dynamic factor in rehabilitation trajectories. In our study, SOC at baseline and increases in SOC over time were associated with better rehabilitation outcomes. Clinically, these findings highlight SOC as a potentially important factor associated with rehabilitation outcomes across patient groups.

## Supplementary Material


