# Supplementary material for: SENSE OF COHERENCE AND REHABILITATION OUTCOMES: A 3-YEAR LONGITUDINAL STUDY
Source: J Rehabil Med. 2026 Sep 29;58:46229. doi: 10.2340/jrm.v58.46229 (PMC13633501; doi:10.2340/jrm.v58.46229)
Supplement: Supplementary file 1 [file JRM-58-46229-s1.pdf]

Table SI. [Prediction model for SOC-13 at baseline]

Abbreviation: CI = 95 % confidence interval.

		Univariable models			Full model			Final model		
**Characteristic**	**N**	**beta**	**95% CI**	**p-value**	**beta**	**95% CI**	**p-value**	**beta**	**95% CI**	**p-value**
Age at baseline (years)	601	0.24	0.16, 0.31	**<0.001**	0.07	0.00, 0.15	**0,06**	0.07	0.00, 0.15	0.065
Sex	601			**<0.001**			**0,01**			**0.016**
female		Ref.	—		Ref.	—		Ref.	—	
male		4.2	2.3, 6.2		2.4	0.58, 4.3		2.3	0.42, 4.1	
Level of education	599			**<0.001**			**0,01**			**0.007**
primary school		Ref.	—		Ref.	—		Ref.	—	
upper secondary school		4	1.3, 6.6		1.7	-0.71, 4.0		1.7	-0.63, 4.0	
University		5.6	2.7, 8.5		3.7	1.2, 6.2		3.8	1.3, 6.3	
Marital status	599			**<0.001**			**0,061**			0.1
unmarried		Ref.	—		Ref.	—		Ref.	—	
married		5.5	2.8, 8.2		2	-0.46, 4.5		1.8	-0.62, 4.2	
Widowed		9.1	4.9, 13		5.7	1.6, 9.9		5.2	1.1, 9.3	
divorced		3.5	0.20, 6.8		2.4	-0.55, 5.4		2.1	-0.81, 5.0	
Referral diagnoses	601			**0.046**			>0.9			>0.9
Musculoskeletal		Ref.	—		Ref.	—		Ref.	—	
Circulatory		3.4	0.81, 5.9		-0.71	-3.2, 1.8		-0.79	-3.3, 1.7	
Neurology		2.4	-1.2, 5.9		-1.1	-4.1, 1.9		-1.1	-4.1, 1.9	
Neoplasms		-2.1	-6.4, 2.2		-0.01	-3.7, 3.7		-0.49	-4.1, 3.2	
Other		1.4	-1.2, 4.1		-0.82	-3.1, 1.5		-0.66	-3.0, 1.6	
Multimorbidity	596			0.2			0,5			
0		Ref.	—		Ref.	—				
1-2		0.47	-3.9, 4.8		-0.9	-4.4, 2.6				
>2		-1.3	-5.8, 3.1		-1.7	-5.4, 2.0				
EQ-VAS baseline	593	0.24	0.19, 0.29	**<0.001**	0.07	0.01, 0.13	**0,019**	0.07	0.02, 0.13	**0.012**
SF36-MCS baseline	558	0.6	0.52, 0.67	**<0.001**	0.53	0.44, 0.61	**<0.001**	0.53	0.45, 0.61	**<0.001**
SF36-PCS baseline	558	0.1	0.00, 0.21	**0.054**	0.1	-0.02, 0.21	0,11	0.1	-0.02, 0.22	0.1

Abbreviation: CI = Confidence Interval
